# Training on Working Memory and Inhibitory Control in Young Adults

**DOI:** 10.3389/fnhum.2016.00588

**Published:** 2016-11-18

**Authors:** Maria J. Maraver, M. Teresa Bajo, Carlos J. Gomez-Ariza

**Affiliations:** ^1^Department of Experimental Psychology – Research Center for Mind, Brain and Behavior, University of GranadaGranada, Spain; ^2^Department of Psychology, University of JaenJaen, Spain

**Keywords:** executive control, cognitive training, working memory, inhibitory control, plasticity, transfer

## Abstract

Different types of interventions have focused on trying to improve Executive Functions (EFs) due to their essential role in human cognition and behavior regulation. Although EFs are thought to be diverse, most training studies have targeted cognitive processes related to working memory (WM), and fewer have focused on training other control mechanisms, such as inhibitory control (IC). In the present study, we aimed to investigate the differential impact of training WM and IC as compared with control conditions performing non-executive control activities. Young adults were divided into two training (WM/IC) and two (active/passive) control conditions. Over six sessions, the training groups engaged in three different computer-based adaptive activities (WM or IC), whereas the active control group completed a program with low control-demanding activities that mainly involved processing speed. In addition, motivation and engagement were monitored through the training. The WM-training activities required maintenance, updating and memory search processes, while those from the IC group engaged response inhibition and interference control. All participants were pre- and post-tested in criterion tasks (*n-*back and Stroop), near transfer measures of WM (Operation Span) and IC (Stop-Signal). Non-trained far transfer outcome measures included an abstract reasoning test (Raven’s Advanced Progressive Matrices) and a well-validated experimental task (AX-CPT) that provides indices of cognitive flexibility considering proactive/reactive control. Training results revealed that strongly motivated participants reached higher levels of training improvements. Regarding transfer effects, results showed specific patterns of near transfer effects depending on the type of training. Interestingly, it was only the IC training group that showed far transfer to reasoning. Finally, all trained participants showed a shift toward a more proactive mode of cognitive control, highlighting a general effect of training on cognitive flexibility. The present results reveal specific and general modulations of executive control mechanisms after brief training intervention targeting either WM or IC.

## Introduction

Executive Functions (EFs) refer to a variety of cognitive and brain mechanisms thought to be in charge of regulating the dynamics of human cognition and behavior in changing environments (Burgess, 1997; Smith and Jonides, 1999; Miyake et al., 2000; Jurado and Rosselli, 2007). In an influential empirical work, Miyake et al. (2000; see also Miyake and Friedman, 2012) used latent variables analyses to show that, despite their unity indicated by shared features, three different EFs emerged from performance in a variety of tasks: (i) *Switching*, which involves shifting flexibly between tasks or mental sets; (ii) *Inhibitory Control* (IC), which refers to overriding dominant or prepotent responses; and (iii) *Updating* of information maintained in Working Memory (WM). WM is usually defined as a cognitive system for temporarily storing and managing information that is necessary for undertaking complex cognitive tasks, and it is thought to play a key role in guiding goal-oriented behavior and novel problem solving (Braver et al., 2008; Unsworth, 2010; Wiley and Jarosz, 2012). As WM is thought to have limited capacity, the ability to update and disengage from information in this system is considered a core component of cognitive control and self-regulation (Miyake and Shah, 1999; Braver and Cohen, 2001).

Although there is some disagreement over the exact nature of EFs and their precise neural substrates (Miyake et al., 2000; Braver and Cohen, 2001; Kane and Engle, 2002; Banich, 2009), substantial evidence supports the fact that EFs play an essential role in learning and academic achievement (Bull and Scerif, 2001; St Clair-Thompson and Gathercole, 2006), knowledge acquisition (Blair and Razza, 2007; Danielsson et al., 2010), metacognition (Fernandez-Duque et al., 2000) as well as emotional and self-regulation (Barkley, 2001; Hofmann et al., 2012). The large role that EFs seem to play in efficient cognition and in successful behavior regulation has led researchers to develop interventions aimed at improving executive functioning, even in the short term. Brain plasticity is at the basis of the proposal that cognitive functioning can be enhanced by means of training. The basic idea is that during cognitive training, participants repeatedly activate neural regions involved in the training tasks (Olesen et al., 2004; Buschkuehl et al., 2012; Hussey and Novick, 2012; Hsu et al., 2014) enhancing, thus, the cognitive function supported by the specific neural region. As a consequence, training effects would generalize and transfer to non-trained tasks that also involve the targeted training domain, and the underlying trained brain areas (*near transfer*) (Lee et al., 2007; Thorell et al., 2009; Borella et al., 2014; Beauchamp et al., 2016). Furthermore, training effects could go beyond the trained domain and show benefits in measures considerably different from the training task, as long as they were associated with the trained process and shared comparable neural circuits (*far transfer*) (Jaeggi et al., 2008; Borella et al., 2010; Loosli et al., 2012; Dahlin, 2013). Similarly, at the behavioral level, transfer effects could be expected in potentially related cognitive functions, and lead to enhanced performance in a variety tasks that, although untrained, share the same cognitive mechanism than the targeted trained processes (Morrison and Chein, 2011). Although plenty of studies have found near transfer effects after training WM, IC, or attention, far transfer effects are still limited and inconclusive (Thorell et al., 2009; Spierer et al., 2013; Sprenger et al., 2013; Enge et al., 2014; Schwaighofer et al., 2015; Melby-Lervåg et al., 2016).

Training studies differ in the type of EFs targeted by the training tasks. WM has traditionally been the target for many cognitive training programs due to its well-known central role in cognition and its relationship with high-level abilities (Klingberg et al., 2005; Morrison and Chein, 2011; Jaeggi et al., 2013). Several studies have demonstrated positive effects of WM training in different age groups (Borella et al., 2010; Söderqvist et al., 2012; Jaeggi et al., 2013) with transfer to trained and untrained domains such as mathematical performance (Dahlin, 2013; Bergman-Nutley and Klingberg, 2014), reading abilities (Chein and Morrison, 2010; Dahlin, 2010; Loosli et al., 2012; Karbach et al., 2015), or reasoning and fluid intelligence (Klingberg et al., 2005; Borella et al., 2010; Jaušovec and Jaušovec, 2012; Au et al., 2014; but see Chooi and Thompson, 2012; Harrison et al., 2013; Redick et al., 2013 for failures to find far transfer effects after WM training; and Melby-Lervåg and Hulme, 2013; Bogg and Lasecki, 2015; Schwaighofer et al., 2015; Dougherty et al., 2016 for reviews).

Some other studies have focused on training IC processes (Spierer et al., 2013). Although several of these studies have failed to find behavioral transfer effects after training IC (Thorell et al., 2009; Berkman et al., 2014; Enge et al., 2014), others have found positive near and far transfer effects after task-switching training across the lifespan of healthy individuals (Karbach and Kray, 2009), training-related benefits in fluid intelligence scores in children after executive control training (Rueda et al., 2005, 2012; Liu et al., 2015), and near transfer effects in normal developing children (Dowsett and Livesey, 2000) or with executive control deficits (Kray et al., 2012). In addition to the behavioral effects, brain activity studies have reported different activation patterns in the brain network associated with IC: namely, increased activation in the right inferior frontal gyrus after training response inhibition in young adults (Berkman et al., 2014); or a more adult-like pattern of EEG markers (dorsolateral prefrontal negativity linked to the anterior cingulate gyrus) in 6-year-old children after a 5-day training with tasks involving conflict resolution (Rueda et al., 2005).

While, with some exceptions, studies focusing on either WM or executive control training show transfer effects (see Karbach and Verhaeghen, 2014 for a meta-analysis with studies that trained WM, switching and IC in older adults), to date very few studies have directly compared the effects of WM and IC training across tasks (see Thorell et al., 2009 for a comparison between WM and IC training in preschoolers). Thus, the main aim of the present study was to directly compare near and far transfer effects of two different training programs targeting either WM or IC processes. The direct comparison of these two types of programs is theoretically interesting since, according to some proposals, WM and IC seem to represent two separate EFs and may therefore have separate effects (Miyake et al., 2000). In addition, we also aimed to carefully control some factors that have been subject to criticism in previous training studies.

As mentioned, despite the studies showing positive results after training in young adults, its effectiveness is still controversial and far transfer effects are not always obtained (Schwaighofer et al., 2015; Melby-Lervåg et al., 2016). Results stemming from different training studies need to be carefully interpreted with special attention to methodological differences that could account for the diversity of findings. Thus, for example, training procedures targeting specific cognitive abilities (such as WM or IC) allow for more restricted attributions on training-derived transfer effects (Borella et al., 2010; Rueda et al., 2012; Jaeggi et al., 2013) than complex procedures that include multiple cognitive domains (memory, attention, IC, reasoning, etc), which seem to be less specific, and often yield more limited transfer effects (Schmiedek et al., 2010; Baniqued et al., 2014, 2015; Dovis et al., 2015; Hardy et al., 2015). Moreover, although many studies have used single training tasks (Rueda et al., 2005; Loosli et al., 2012; Carretti et al., 2013; Jaeggi et al., 2013), the potential generalization of the training might be enhanced by the use of different tasks recruiting the particular targeted process. Switching between multiple tasks targeting the same process during training might promote cognitive flexibility by adapting general processes and strategies, and thus, preventing the use of very specific task-strategies that would more likely be implemented when training is based on just one single task (Schmidt and Bjork, 1992; Bherer et al., 2005; Dahlin et al., 2008; Schwaighofer et al., 2015). Finally, the presence and type of control groups is an essential requirement to dissociate the effectiveness of training (Mohr et al., 2009; Dougherty et al., 2016; Melby-Lervåg et al., 2016). Thus, passive control groups (PC) may allow researchers to keep track of simple practice effects, while active control (AC) groups may enable them to uncover the specificity of different cognitive training procedures by maintaining similar levels of motivation and reducing the possibility of placebo effects (Boot et al., 2013; Dougherty et al., 2016).

Hence, in the present study we explored potential transfer effects of two executive-control training programs in young adults. We used a procedure that attempted to maximize generalization (by using multiple activities within each trained process) and to control for practice and motivation (by including active and passive control groups and by capturing motivational variables during training). Specifically, we compared a group of participants trained in working memory (WMT) to a group trained in inhibitory control (ICT). Both groups were trained with three different training activities during six sessions spread across 2 weeks. Importantly, the training procedures were adaptive and increased executive control demands. We included passive (PC) and active (AC) control groups in the study. AC performed the same training protocol as their experimental counterparts, but they engaged in activities that relied on perceptual abilities and progressively increased their speed demands, without increments in executive load (Peng et al., 2012; Takeuchi and Kawashima, 2012; Lawlor-Savage and Goghari, 2016). The batteries of training activities used here were designed from the Cognitive Training Program of the University of Granada (PEC-UGR^1^), which included a number of tasks that could be adapted and combined. We designed these batteries considering both the neural basis of the cognitive processes underlying the activities and the logic of the experimental procedures traditionally used to evaluate executive control. As for the ICT group, the training included versions of (i) the Stroop task in which participants had to select coins/numbers contained in congruent or incongruently sized bags; (ii) the Conflict resolution task, where a sample of animals was presented and participants were asked to search for a target match from a set of distractors displaying congruent/incongruent shaped/colored animals; and (iii) the Go/No-Go and Stop-Signal tasks, in which participants had to respond to matching shapes (a robot and a screw), and stop their response when faced with a rusted-looking shape. Regarding WMT, participants performed versions of (i) the *n-*back task, in which participants had to monitor sequences of open/closed windows from a six-window display presentation, and press a key whenever the open window was the same as the window *n* trials back; (ii) WM updating, that consisted of the serial presentation of objects of different sizes that were introduced in boxes. Participants were asked to recall the 2 to 6 largest/smallest elements from the series; and (iii) Dual Span tasks, in which participants were asked to recall the shape and color of an increasing number of animals and then ask to select the animal that matched one of the study animals from a set of distractors. Participants were evaluated before and after training with two criterion tasks (*n*-back and Stroop), with near transfer WM (Operation Span) and IC (Stop-Signal) tasks, as well as with far transfer non-verbal reasoning (Raven’s Advanced Progressive Matrices). In addition, we included a far transfer task (AX-CPT; the AX version of the Continuous Performance Test) to explore whether WM and IC training might change the adjustment of distinct executive control strategies (proactive versus reactive), which have been proposed to support cognitive flexibility^2^ (Braver et al., 2009; Burgess et al., 2011; Braver, 2012).

Also of relevance, in the present study we also aimed to explore the role of individual differences on training and transfer performance. This represents a recent and unexplored issue that may be important in predicting the benefits of training (Könen and Karbach, 2015). In this sense, previous studies have already reported that at baseline, reasoning predicts training achievement (Bürki et al., 2014). Furthermore, individuals’ improvement during training has been shown to be a relevant predictor of transfer effects in young adults (Jaeggi et al., 2011) as well as in children and older adults (Zinke et al., 2013; Wang et al., 2014). Thus, in order to explore the potential role of individual differences in training success, predictors of training improvement and transfer gains were analyzed (Könen and Karbach, 2015).

Based on the key assumption that generalization to non-trained tasks could occur whenever there is cognitive and neural overlap between the trained processes and those engaged in the outcome measures (Woodworth and Thorndike, 1901; Persson and Reuter-Lorenz, 2008; Hussey and Novick, 2012), we expected the two experimental groups to exhibit differential and specific enhanced post-training performance (for related findings, see Chein and Morrison, 2010; Foy and Mann, 2014). Thus, we expected that, after training, the WMT group would outperform the ICT group on the *n*-back and Operation Span tasks, which involved WM maintenance demands. On the other hand, due to the greater reliance on conflict resolution for the ICT group than for the WMT group, we predicted better performance after IC training in the Stroop and Stop-Signal tasks relative to the WMT group.

Regarding the active control group, which went through progressive response speed demands, we expected benefits in response times after training. Processing speed, even at a low demand level, may lead to changes in performance mainly driven by the fact that participants’ responses could become faster after the training (Peng et al., 2012; Takeuchi and Kawashima, 2012; Lawlor-Savage and Goghari, 2016).

As for the AX-CPT, which provided an index of the control strategy deployed by the participants, we hypothesized that the two executive control-training programs would make participants more dependent on proactive control relative to control conditions. This hypothesis is based on the assumption that the high executive control demands of both training programs would encourage participants to focus on contextual cues and, hence, to enhanced reliance on cue processing (rather than probe processing) on the AX-CPT task. If so, both types of training would lead to maximize the typical proactive strategy deployed by young healthy adults. However, we also expected the WM training, which specifically focuses on monitoring and maintenance, to have a stronger impact on proactivity than IC training.

Finally, on the basis of either the close relationship between matrix problem resolution, WM (Colom et al., 2004; Friedman et al., 2006; Harrison et al., 2015) and executive control (Dempster and Corkill, 1999; Engle and Kane, 2004; Jarosz and Wiley, 2012; Shipstead et al., 2015) and the results of some previous training studies (Jaeggi et al., 2008; Karbach and Kray, 2009; Rueda et al., 2012; Au et al., 2014), we expected to find better post-training performance in the reasoning test in the two experimental groups than in the active and passive control conditions.

## Materials and Methods

### Participants

Participants were recruited via physical ads in the University of Granada requiring the fulfillment of the following conditions: (i) be aged between 18 and 30 years old; (ii) not to have any major medical or psychological condition; (iii) be committed to undertake at least four experimental sessions in the lab, which could be extended to 10. One hundred and twelve undergraduate students were selected to take part in the present study (*M*_age_ = 20.51 years; *SD*_age_ = 1.74; range = 18 – 25; 83 females). After pre-testing, they were randomly assigned to one of the four groups making up the study: ICT, (*N* = 32; *M*_age_ = 20.41 years; *SD*_age_ = 1.88; 23 females), WMT (*N* = 32; *M*_age_ = 20.31 years; *SD*_age_ = 1.57; 23 females), active control (AC, *N* = 24; *M*_age_ = 20.75 years; *SD*_age_ = 1.32; 18 females), or passive control (PC, *N* = 24; *M*_age_ = 20.67 years; *SD*_age_ = 2.16; 19 females). There were no significant differences either in age (*p* = 0.76; $\eta_{p}^{2}$ = 0.01) or in gender distribution (*p* = 0.92; $\eta_{p}^{2}$ = 0.00). At the end of the study, the participants were economically compensated for their involvement. None of the participants withdrew from the study although they were informed they could do so if they wished. This study was approved and carried out in accordance with the recommendations of the Research Ethics Committees of the University of Granada, with written informed consent from all subjects. All participants were provided with information about the study and gave written informed consent in accordance with the Declaration of Helsinki (World Medical Association, 2013).

### Procedure

The cognitive training schedule consisted of two (pre- and post-training) testing sessions and six training sessions distributed over 2 weeks, with three training sessions per week. Therefore, the total length of the study extended for approximately 4 weeks. In the testing sessions all of the participants were evaluated for: (i) criterion tasks (*n*-back and Stroop); (ii) WM (Operation Span) and IC (Stop-Signal) as near transfer measures; and (iii) adjustment of proactive/reactive cognitive control (AX-CPT) and abstract reasoning (Raven’s Advanced Progressive Matrices) as far transfer measures. We created two random task orders for evaluation that were counterbalanced across participants. The training and active control groups engaged in three different activities during each session (20 min per activity). The order of the activities in each training session was also counterbalanced over all participants. The resulting total training time for each activity was 120 min. The passive control group only performed the evaluation sessions and continued with their regular college activity during the 2 weeks between pre- and post-training.

Participants worked in individual cabins although an experimenter continuously supervised the procedure and was available to attend to any request. Every two training sessions participants were to complete a motivation questionnaire (Alonso-Tapia and de la Red Fadrique, 2007; Colom et al., 2013) in which they were asked for their: (i) involvement in the program; (ii) perceived difficulty of the activity levels; (iii) perceived challenge of improving over the levels; and iv) expectations for their achievement. They had to rate each of the four statements on a scale ranging from 0 (very low) to 10 (very high). In the last training session, they were asked for a general evaluation of the training program and their satisfaction with the experimental procedure.

### Executive Control Training

We used the online training program from the University of Granada (PEC-UGR) that included different game-like activities organized in levels of increasing difficulty. Training difficulty was adaptive in order to maintain activities as a constant challenge (Klingberg et al., 2005; Brehmer et al., 2011; Karbach et al., 2015). Also, participants received feedback on whether their performance was correct or not (Katz et al., 2014). Activity levels were built up over runs of trials. Whenever participants succeeded in three runs they went forward to the next level and if they failed two runs, they went back to the previous level. Details of each of the three activities per training group are detailed below.

#### Inhibitory Control Training

##### Stroop-like

This activity was modeled on the Steinhauser and Hübner’s (2009) complex Stroop task, which involved both conflict resolution and switching. The task was implemented in a scenario where bags of different sizes containing amounts of money had to be put into a treasure chest. Participants had to select the bag with the largest (gold in color) or the smallest (silver in color) number of items, with the number of bags increasing over the levels. The size of the bags could be either congruent or incongruent with the amount inside. An example of a congruent trial is one in which the stimuli were a big bag containing seven golden coins (correct choice) and a small bag containing five golden coins. In an example of an incongruent trial the stimuli could be a small bag containing six golden coins (correct choice) and a big bag containing three golden coins. Difficulty increased by changing the ratio of congruent/incongruent trials (0; 0.25; 0.50; 0.75), so that the larger the proportion of congruent trials, the harder the choice for incongruent trials. At higher levels, switching was manipulated by changing the color of the items from gold to silver between trials within the same round. Times to respond and inter-stimuli intervals were also progressively reduced with each level. The dependent variable was a relative index of conflict resolution [(RT in incongruent trials – RT in congruent trials)/RT in congruent trials].

##### Conflict resolution task

The scenario of this activity was an ocean where a sample of sea animals was displayed in the upper part of the screen and a group of animal buttons was shown in the lower part. The buttons set size was always sample *n* + 1 and it was progressively increased over the levels from 2 to 6. Participants had to select, as fast as possible, the animal of the buttons that had the same shape and color as one of the animals in the sample (match trials). If there was an animal whose shape matched but the color did not, they had to click on the different button (no-match trials). An example of match trial could be one in which the sample stimuli included “blue turtle – yellow starfish – brown crab” and the button choices included “pink turtle – yellow starfish (correct choice) – red crab – gray dolphin.” On the other hand, a no-match trial could be one displaying as sample “blue turtle – yellow starfish – brown crab” and the button choices containing “pink turtle – green starfish – red crab – gray dolphin (correct choice).” The percentage of match trials was manipulated (0.25; 0.5; 0.75) so that the higher this ratio, the stronger the tendency to respond. Difficulty was also manipulated with the similarity of the color between the sample and the choice of the buttons. When colors were limited (a different color between the target and the possible options), the choice got harder since the color of the distractors had to be inhibited. The time to respond and inter-stimuli intervals were also reduced over the levels. The parameter distribution across the levels was manipulated following the procedures used in Rueda et al. (2005, 2012). As in the previous activity, the dependent variable was the score in the relative index of conflict.

##### Go/No Go-like

This was a matching-to-sample activity based on the shape of the items: a robot was the target and a screw was the sample. Participants had to respond when the shape of the robot and the screw matched (Go trials: i.e., a squared robot and a squared screw on its top) and inhibit their response when the shapes did not match (No-Go – shape trials: i.e., a circled robot and a squared screw on its top). At higher levels, there was an extra difficulty because the response had to be also inhibited when the screw was rusted (No-Go – color trials: i.e., a squared robot and a rusted squared screw on its top), even if its shape matched that of the robot. The proportion of Go trials (0.10; 0.20; 0.50; 0.80; 0.90) was manipulated together with the additional No-Go color trials ratio (from 0 to 0.30). The higher the proportion of Go trials, the stronger the tendency to respond with greater IC being required to succeed. The manipulation of the parameters was conducted following similar procedures regarding Go/No Go trials proportion (Benikos et al., 2013b) and reaction times deadlines (Benikos et al., 2013a). As in the previous activities, the maximum time to respond was reduced when levels increased (Benikos et al., 2013a). In this case, false alarms and omission errors were the dependent variables.

#### Working Memory Training

##### N-back

Participants had to monitor, maintain and continuously update the items throughout a sequence of elements. Participants were presented with a six-window house and had to detect coincidence between positions (opening/closing of the windows), sounds, or the combination of both modalities. They had to give their response pressing a button whenever the position of the opening window, its sound or both, matched the one that was presented as *n* positions-back in the sequence. Increments in *n*-back (from 1 to 8) were implemented after participants had completed the *n*-back level with single (position or sound) and dual (position plus sound) modality levels. As for the dependent variables, we considered the achieved *n*-back level and the sum of errors in each session.

##### WM search

This was a matching-to-sample activity based on the shape and color of the items sequentially displayed: animals on one screen as the sample, and a group of animal buttons after a retention interval. Participants were presented with a matrix to be maintained in memory composed of animals with different shapes and colors displayed in an open field (i.e., memory matrix, “brown bear – red eagle – purple snake”). After a retention time of 5000 ms, participants performed a memory test in which they had to select as fast as possible the animal on the buttons that had the same shape and color of one of the previously retained animals (i.e., button choices, “orange bear – red eagle (correct choice) – yellow snake – blank button”). If none of the animals on the buttons had the same shape and color, they had to select the blank button (i.e., button choices, “orange bear – green eagle– yellow snake – blank button (correct choice)”). The number of to-be-maintained items increased from 1 to 8 over the levels. The number of elements recalled (set size) was the dependent variable.

##### WM updating

This task was adapted from the word updating task from Palladino and Cornoldi (2001). Participants were presented with a group of numbered boxes. Items of different categories (food, objects, animals, or clothing) were sequentially displayed. For each trial, items from only one category were relevant and introduced into the boxes (i.e., animals). Participants were asked to recall the larger (or smaller) element(s) by selecting the box or boxes in which they were introduced (i.e., Rule*:* recall the smallest animal; Items presented*:* apple – cat – trousers – bee (correct choice) – chair – elephant). Maintenance and updating in WM were involved in this activity. The memory load was manipulated by increasing the number of elements to recall (from 1 to 7), the number of distractors that belong to the target category (from 1 to 7), and the number of distractors from different categories (from 2 to 20). The program randomly changed the rule from big to small keeping an equal proportion of the trials within a level. The dependent variable was the number of items successfully recalled.

#### Active Control

##### Speed comparison

In this matching-to-sample activity, participants were presented with a group of sea animals in the upper half of the screen and another group of animals in the lower half, and they were asked to find as fast as possible which animal in the lower part was present in the upper part of the screen. In all of the trials, the target was presented in the sample, which increased from 2 to 6. Times were reduced within each sample size, so that whenever one element was added to the sample the time to respond started at a higher level at the beginning and was progressively reduced. Response time was the dependent variable.

##### Speed visual search

For this speed of processing task, participants were presented with a plate of soup with 10 elements (digits and letters) and they had to find one element contained in the soup out of four different possible options. The number of elements to be found and the possible options remained constant so that the difficulty of the levels was only determined by the speed of the responses over the levels. Average reaction time per session was the dependent variable in this case.

##### Speed categorization

This activity required participants to categorize groups of figures while progressively reducing the time to do so over the levels. Participants saw three groups of figures and two boxes to classify them according to different rules (size, color, shape, or quantity). The rule to be applied for categorizing them was always displayed in the upper left corner of the screen so that it trained the response time throughout the levels. As in the two previous activities, we considered reaction time as the dependent variable.

### Transfer Tasks

#### Stroop

The scenario of this task was similar to the one used for training, where participants were presented with different-sized bags and they had to select one with the largest (or smallest) amount inside. The bags were either congruent or incongruent in size. The switching component was manipulated by changing the color of the coins and the consequent response rule from the largest (gold) to the smallest (silver). The number of bags presented (from 2 to 7), the proportion of incongruent trials (0, 0.25 or 0.50), the proportion of switching trials (0, 0.25, 0.50, 0.75), the inter-stimuli interval (from 1500 to 600 ms) and the maximum time to respond (from 3000 to 3600, this increased as a function of the number of bags) was manipulated across blocks of trials (levels). Inter-stimuli intervals were designed considering the average ITI used in the study by Steinhauser and Hübner (2009); the maximum and minimum intervals limited a wider range than the one parametrized for the training so that enough room was left to observe a possible benefit in response time. The dependent variable (conflict score) was calculated as a relative index from (Incongruent trials RT – Congruent trials RT)/Congruent trials RT, for hits. Stimuli were presented randomly both in pre- and post-training testing sessions.

#### *N-*back

In this WM task, participants had to retain the spatial position of a sequence of elements over nine blocks of increasing memory load. Participants had to give a response any time an element matched the position of an element presented *n* (from 1 to 8) position-back. The length of the sequence in a block increased parallel to the memory load, from 6 to 20. The maximum time to respond and the inter-stimuli interval was, respectively, 2000 and 1000 for the first four blocks, and 1500 and 800 for the four last ones. *N*-back level and errors (omissions and commissions) were considered as dependent variables. The order of the stimuli in the sequence was randomized in pre- and post-testing.

#### Stop-Signal

We used this task of response inhibition with the standard parameters of the software STOP-IT (Verbruggen et al., 2008). Participants had to respond with the keyboard as fast as possible to two different stimuli (circles or square) presented in the center of the screen. In 25% of the trials, participants faced an auditory stop-signal (750 Hz, 75 ms) that was presented briefly after the visual stimuli onset and required the response to the current stimulus to be withdrawn. The task comprised of 32 practice trials and three blocks with 64 experimental trials each. The trials were displayed on a black screen and were composed of a 250 ms fixation point (white +), the stimuli presentation (a white square or circle) during 1250 ms and a fixed inter-stimuli interval of 2000 ms. Stimuli were randomized in pre- and post-testing, and in all cases the stop signal was presented with a variable stop-signal delay (SSD). Although initially it was set to 250 ms, it was continuously adjusted. When the inhibition was successful it was reduced 50 ms and if not, increased in 50 ms, so that according to the performance the software tried to maintain a stopping probability of 50%. We considered the Stop-Signal Reaction Time (SSRT) as a measure of motor inhibition efficiency (Verbruggen and Logan, 2008; Verbruggen et al., 2008; Morales et al., 2013).

#### Operation Span (O-Span)

We used the Spanish adapted version of the procedure developed by Turner and Engle (1989) (Turner and Engle, 1989; Tokowicz et al., 2004; Redick et al., 2012). It was a dual memory span task that required participants to verify mathematical operations while trying to remember sets of words of increasing set sizes. Each trial was composed of a simple solved mathematical equation [i.e., (14/2) + 2 = 9] presented for 3750 ms that participants had to verify and mark as correct or not by pressing one out of two keys on the keyboard. Afterward, a word was presented for 1250 ms to be maintained in memory. Operation-word pairs were presented in increasing set sizes from 2 to 6. After each set, participants had to recall and type the words. While the order of recall was not important, they were told to avoid writing the last word presented first in order to prevent recency effects. The task comprised of 18 trials (three trials per set size) and the testing procedure was repeated until the end. We developed parallel versions of the task by randomizing the order of the stimuli presented that were counterbalanced across sessions and participants. Two parallel versions were created and counterbalanced for pre- and post-testing by randomizing the equation-word pairing. Special care was taken to avoid a similar pairing set size distribution between the two versions.

#### AX-CPT

We used the same version of the task as Morales et al. (2013) did to explore the adjustment of proactive/reactive cognitive control. In each trial participants were presented with five letters for 300 ms each (cue – three distractors – probe) in the center of a black screen, with a fixed inter-stimuli interval of 1000 ms. Cue and probe stimuli were presented in red font while distractors were presented in white. Participants were instructed to respond “yes” whenever they saw an A in the first position (cue) followed by an X in the fifth position (probe). Participants were asked to respond “no” to any other cue-probe combination and to the distractors (items in positions 2 to 4). The task was composed of a 10 trials practice phase and an experimental block of 100 trials, which were presented randomly both in pre- and post-testing. The target trials (AX) were the most frequent ones (70%) and the rest of the trials (cue – distractor: AY; distractor – probe; BX or neither cue nor probe: BY) occurred in a 10% of the remaining cases. Proactive and reactive control adjustment can be assessed by considering the proportion of errors in AY and BX type trials (Braver et al., 2009; Morales et al., 2013; Chiew and Braver, 2014).

#### Raven’s Advanced Progressive Matrices (RAPM)

We used the computerized version of the set II of this test as a standardized measure of fluid intelligence (Raven, 1990). Participants had to solve visual analogy problems of increasing difficulty. A 3 × 3 matrix of patterns was presented and they had to a missing pattern of a matrix, from eight different response alternatives. We counterbalanced two parallel versions of the test over sessions with 18 matrices for the pre- and post-testing as used by Jaeggi et al. (2013). Participants had to complete the task as fast and accurately as possible with a 20 min time restriction. The dependent variable was the proportion of correct matrices answered and the reaction times of the hits.

## Results

### Training Effects

To determine the significance of the training improvement in each activity, we compared the performance in the first training session with that of the final training session (sixth session). Thus, for all tasks repeated-measures analyses of variance (ANOVAs) were conducted on the specific dependent variables for the task (conflict score, errors, reactions times, or memory load) with training session (first vs. sixth) as the within-subject independent variable.

#### ICT Group

For the Stroop-like task, the reaction times from 20 participants (10 from ICT and 10 from WMT) were not registered due to a software coding error and consequently they could not be included in the analyses. The ANOVA yielded a reliable difference in the relative conflict effect [(incongruent-congruent)/congruent hits RT] from the first to the last training session [*M*_s1_ = 0.52, *SD*_s1_ = 0.24; *M*_s6_ = 0.33, *SD*_s6_ = 0.19; *F*(1,21) = 5.94; *p* = 0.02; $\eta_{p}^{2}$ = 0.22]. The conflict effect was also reduced from the first to the last training session in the Conflict resolution task, although the difference did not reach statistical significance [*M*_s1_ = 0.48, *SD*_s1_ = 0.34; *M*_s6_ = 0.39, *SD*_s6_ = 0.25; *F*(1,31) = 1.71; *p* = 0.20; $\eta_{p}^{2}$ = 0.05]. For the Go/No-Go task we analyzed both omission errors and false alarms. The results of these analyses showed that participants reduced their average omission errors [*M*_s1_ = 3.50, *SD*_s1_ = 2.68; *M*_s6_ = 1.43, *SD*_s6_ = 2.01; *F*(1,31) = 13.11; *p* < 0.01; $\eta_{p}^{2}$ = 0.30], while the reduction of false alarms was not reliable [*M*_s1_ = 3.90, *SD*_s1_ = 3.50; *M*_s6_ = 3.25, *SD*_s6_ = 2.70; *F* < 1; *p* = 0.40; $\eta_{p}^{2}$ = 0.02].

#### WMT Group

For all the WM-training tasks (*n*-back, WM search and WM updating), we compared the memory set size recalled from the first to the last training sessions. The increment in set size recalled was statistically significant for all the three activities trained: *n*-back [*M*_s1_ = 1.13, *SD*_s1_ = 0.17; *M*_s6_ = 2.60, *SD*_s6_ = 0.57; *F*(1,31) = 190.92; *p* < 0.01; $\eta_{p}^{2}$ = 0.75]; WM Search [*M*_s1_ = 2.21, *SD*_s1_ = 0.17; *M*_s6_ = 4.92, *SD*_s6_ = 1.07; *F*(1,31) = 198.32; *p* < 0.01; $\eta_{p}^{2}$ = 0.76] and WM Updating [*M*_s1_ = 1.00, *SD*_s1_ = 0.06; *M*_s6_ = 3.20, *SD*_s6_ = 0.63; *F*(1,31) = 390.95; *p* < 0.01; $\eta_{p}^{2}$ = 0.86].

#### AC Group

Note that this group did not change the level of executive demands, which was held constant throughout the training sessions. Although, they went forward over levels, so their impression was that they were training, the changes from one level to the next were the progressive reduction of presentation speed and response-time. Hence, we compared the speed of the participants’ responses (ms) from the first to the last session for the three activities. The results of this comparison yielded statistically significant differences for Speed Comparison [*M*_s1_ = 5075.87, *SD*_s1_ = 437.75; *M*_s6_ = 3539.04, *SD*_s6_ = 656.75; *F*(1,23) = 89.62; *p* < 0.01; $\eta_{p}^{2}$ = 0.66]; Speed Visual Search [*M*_s1_ = 22555.57, *SD*_s1_ = 864.64; *M*_s6_ = 3585.23, *SD*_s6_ = 534.92; *F*(1,23) = 8096.82; *p* < 0.01; $\eta_{p}^{2}$ = 0.99] and Speed Categorization [*M*_s1_ = 24987.26, *SD*_s1_ = 62.39; *M*_s6_ = 13731.60, *SD*_s6_ = 194.22; *F*(1,23) = 374.22; *p* < 0.01; $\eta_{p}^{2}$ = 0.89].

#### Training Slopes

**FIGURE 1:**
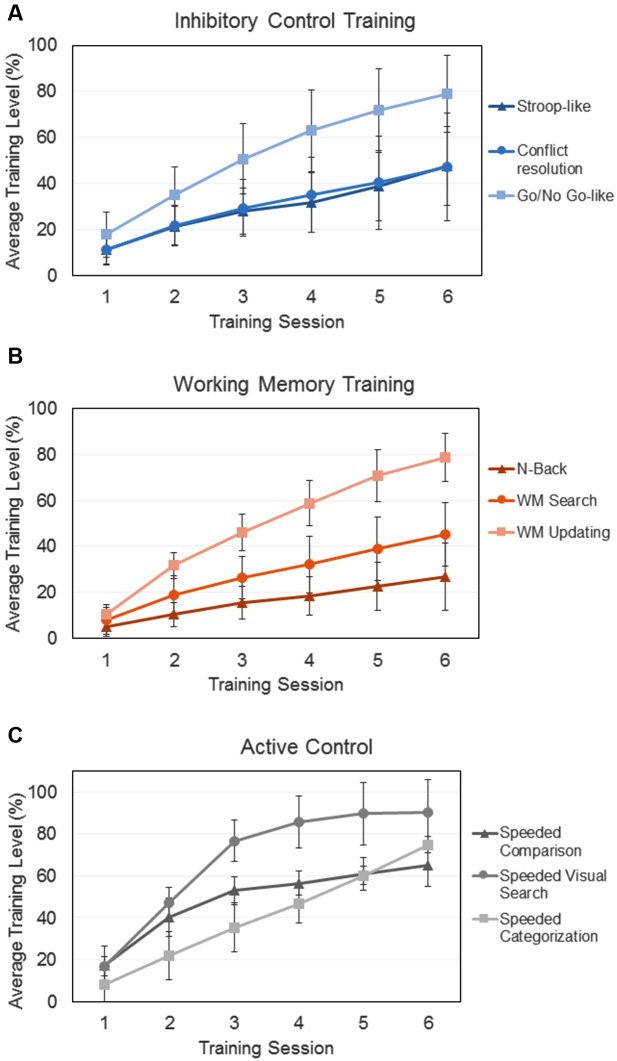
**Training improvement over the six training sessions for the three different groups that complete training procedures:** **(A)** Inhibitory Control Training, **(B)** Working Memory Training, **(C)** Active Control. In all the cases, *y*-axes represent the average relative level achieved in each session and training activity. Error bars represent standard deviations.

The training program PEC-UGR enabled us to create many training levels by using all possible combinations of task parameters (i.e., proportion of congruent/incongruent trials; target-distractor similarity; memory load; response times; etc.). Nonetheless, because the tasks differed in the number of to-be-manipulated parameters, the number of training levels varied across activities. Consequently, in order to put together the trained activities and to compare how far participants from the different groups went in the training, we standardized the level of achievement for each participant by dividing the average level reached in a given activity by the number of levels possible in the activity. Thus, **Figure 1** represents the relative level achieved in each activity and each training session for the three trained groups.

To quantify participants’ training improvement over the six sessions of training, we calculated the slope of a linear regression model using the standardized average level in each training session and activity per participant (Katz et al., 2014; Wang et al., 2014). In order to compare the training achievements of the different groups (**Figure 1**), slopes of the three training tasks for each group were averaged. A one-way ANOVA showed a main effect of group [*F*(2,85) = 16.26; *p* < 0.01; $\eta_{p}^{2}$ = 0.27], as the average slope for the AC (*M* = 12.23; *SD* = 1.34) was significantly larger than the one for the ICT (*M* = 8.68; *SD* = 2.92; *p* < 0.01) and the WMT (*M* = 8.05; *SD* = 1.78; *p* < 0.01) groups. This is consistent with the fact that active control activities were significantly easier that the executive control ones, facilitating the advancement through the activity levels. The slopes of the two experimental training groups did not differ one from each other (*p* = 0.76).

### Correlations at Pre-test

To check for relationships between the cognitive functions tested at baseline, Pearson correlations were run on the pre-test scores for all the participating groups as a whole. These analyses showed that WM-related measures were correlated: those participants with a higher combined score in the O-Span task showed fewer intrusions in the O-Span (*r* = -0.34; *p* < 0.01) and fewer errors in the *n*-back task (*r* = -0.19; *p* = 0.03). Additionally, participants with a larger BSI showed fewer intrusions in the O-Span task (*r* = -0.20; *p* = 0.03).

Finally, RAPM scores significantly correlated with errors in the *n*-back (*r* = -0.21; *p* = 0.10) and with the combined score from the O-Span task (*r* = 0.31; *p* < 0.01).

### Transfer Results

**Table 1** summarizes the descriptive data of the outcome measures, including statistical comparisons for the session effects (pre vs. post) in each of the groups. We also calculated standardized gains subtracting the pre-test scores from the post-test (the opposite for reaction times and errors) and divided by the standard deviation of the entire sample (Colom et al., 2013; Jaeggi et al., 2013; Redick et al., 2013; Borella et al., 2014). One-way ANOVAs were performed for each variable in order to compare standardized gains between the groups. The participants who were excluded at pre-test due to missing data were also excluded from analyses of performance after the training.

**Table 1:** Descriptive statistics for outcome measures: Mean and standard deviations for the outcome measures in the pre- and post-testing.

		Pre-test		Post-test		Pre–Post effects		Standardized Gain	
					
Variables	*N*	*M*	*SD*	*M*	*SD*	*p*	*d*	*M*	*SD*
**Stroop: Conflict effect ((Incongruent – Congruent)/Congruent) hits RT (ms)**					
IC Training	21	0.12	0.20	0.01	0.03	0.02^∗^	0.76	0.61	1.01
WM Training	22	0.14	0.21	0.09	0.14	0.32	0.28	0.25	0.99
Active Control	24	0.04	0.07	0.05	0.04	0.62	-0.17	-0.05	0.50
Passive Control	24	0.04	0.18	0.07	0.30	0.70	-0.12	-0.13	1.72
**N-back: level**					
IC Training	32	1.55	0.82	1.72	0.95	0.28	0.19	0.07	1.05
WM Training	32	1.78	0.83	2.43	0.75	0.00^∗^	0.82	0.75	1.23
Active Control	24	1.95	0.80	2.12	0.94	0.44	0.19	0.19	1.21
Passive Control	24	1.66	0.96	1.41	0.58	0.20	-0.31	-0.28	1.09
**N-back: errors**					
IC Training	32	8.51	4.77	8.51	5.48	1.00	0.00	0.12	1.28
WM Training	32	9.81	4.40	6.34	3.80	0.00^∗^	0.84	0.79	1.26
Active Control	24	9.95	3.74	9.70	2.64	0.76	0.07	0.05	0.93
Passive Control	24	4.62	1.78	4.20	1.44	0.38	0.25	0.09	0.53
**Stop-Signal: SSRT (ms)**					
IC Training	30	280.47	39.02	255.54	53.56	0.00^∗^	0.53	0.54	0.97
WM Training	31	266.29	51.68	254.66	36.19	0.28	0.26	0.25	1.30
Active Control	21	258.96	48.16	244.30	51.59	0.10	0.29	0.32	0.87
Passive Control	22	254.55	39.36	239.30	37.17	0.19	0.39	0.33	1.17
**O-Span Index: Words recalled × Equations hits**					
IC Training	32	0.59	0.10	0.66	0.12	0.00^∗^	0.63	0.52	0.68
WM Training	32	0.59	0.18	0.66	0.19	0.00^∗^	0.37	0.45	0.82
Active Control	24	0.55	0.13	0.60	0.13	0.00^∗^	0.38	0.19	0.28
Passive Control	24	0.58	0.12	0.61	0.10	0.24	0.27	0.18	0.77
**O-Span: Intrusions**					
IC Training	32	2.31	1.71	1.78	1.56	0.10	0.32	0.26	0.87
WM Training	32	2.46	1.81	1.34	1.42	0.00^∗^	0.69	0.55	0.92
Active Control	24	3.00	2.91	2.70	2.31	0.46	0.11	0.14	0.94
Passive Control	24	2.50	1.61	2.87	2.29	0.44	-0.18	-0.18	1.15
**AX-CPT: BSI (AY-BX)/(AY+BX)**					
IC Training	28	0.42	0.13	0.50	0.17	0.01^∗^	0.52	0.48	1.03
WM Training	30	0.39	0.17	0.51	0.14	0.00^∗^	0.77	0.66	1.26
Active Control	23	0.40	0.18	0.45	0.17	0.15	0.28	0.33	1.08
Passive Control	23	0.39	0.19	0.36	0.32	0.72	-0.11	-0.15	2.04
**RAPM: Hits**					
IC Training	32	0.44	0.18	0.51	0.18	0.01^∗^	0.38	0.36	0.77
WM Training	32	0.43	0.20	0.47	0.17	0.34	0.21	0.20	1.18
Active Control	24	0.47	0.20	0.49	0.18	0.44	0.10	0.14	0.91
Passive Control	24	0.48	0.15	0.42	0.17	0.06	-0.37	-0.32	0.76
**RAPM: Hits RT (s)**					
IC Training	32	35.75	12.94	33.87	15.50	0.49	0.13	0.12	0.98
WM Training	32	34.42	16.63	31.38	15.92	0.15	0.18	0.19	0.76
Active Control	24	42.32	17.13	40.51	15.27	0.55	0.11	0.11	0.93
Passive Control	24	34.44	15.55	32.24	16.44	0.48	0.13	0.14	0.95


*Significance *p*-values and effect sizes (Cohen’s *d*) estimators are reported for the Repeated-Measures ANOVAs including session as a within subject variable (pre-test and post-test values) and group as a between-subject effect in each of the four groups. Standardized gains were computed as (Mpost – Mpre)/(SDpre) for hits and as (Mpre – Mpost)/(SDpre) for errors and reaction times.*

#### Stroop

We obtained a relative conflict score from the difference in reaction times between incongruent and congruent trials. There were no pre-test differences between the groups [*F*(3,88) = 1.86; *p* = 0.14; $\eta_{p}^{2}$ = 0.05]. The ANOVA on the standardized gains failed to show a reliable effect of group, *F*(3,88) = 1.76; *p* = 0.16; $\eta_{p}^{2}$ = 0.05. As can be observed in **Table 1**, however, it was only the ICT group that was able to significantly reduce their conflict scores after completing the training.

#### *N*-back

The *n*-back level and the number of errors were considered in this task. There were no differences in the baseline *n-*back level before training [*F*(3,108) = 1.39; *p* = 0.24; $\eta_{p}^{2}$ = 0.03]. However, a one-way ANOVA revealed differences in the number of errors at pre-test [*F*(3,108) = 10.02; *p* < 0.01; $\eta_{p}^{2}$ = 0.21], whereby the PC group committed significantly fewer errors than the other three groups (all *p*s < 0.01). **Table 1** shows that only the WMT group showed a reliable increase in the number of items that could be maintained/updated in WM and a reduction in the number of errors committed after the training. The ANOVA performed on the standardized gains scores revealed a statistically significant effect of group for *n*-back level: [*F*(3,108) = 4.06; *p* < 0.01; $\eta_{p}^{2}$ = 0.10]. *Post hoc* comparisons for the *n*-back level indicated that the only reliable difference was between the PC and the WMT groups (*p* < 0.01) whereas the pairwise comparisons between the remaining groups were not significant (ICT-WMT: *p* = 0.11; ICT-AC: *p* = 1.00; ICT-CP: *p* = 1.00; WMT-AC: *p* = 0.42; PC-AC: *p* = 0.89). In the case of errors, and because we found differences between groups at pre-test, we checked whether there were group differences in the standardized gains as *n*-back errors committed at pre-test were introduced as a covariate. The analysis of covariance (ANCOVA) revealed a reliable effect of the covariate [*F*(3,107) = 59.06; *p* < 0.01; $\eta_{p}^{2}$ = 0.35] but also a significant effect of group [*F*(3,107) = 5.28; *p* < 0.01; $\eta_{p}^{2}$ = 0.13]. Further analyses showed that there was a statistically significant difference between the AC and WMT groups (*p* = 0.01), and between the two control groups (*p* < 0.01). None of the other comparisons showed reliable differences (ICT-WMT: *p* = 0.39; ICT-AC: *p* = 0.89; ICT-CP: *p* = 0.14; WMT-CP: *p* = 1.00).

#### Stop-Signal

We used the software ANALYZE-IT provided by Verbruggen et al. (2008) to determine the impact of training on inhibition. The SSRT is an index of pure response inhibition and the program calculates it by subtracting the SSD from the untrimmed RT mean. Following the criteria of Verbruggen, we removed five participants (one ICT, one WMT, one AC, and two PC participants) from the analysis, since they had an overall probability of responding on stop trials significantly below or above 50% in both pre- and post-test. The groups did not differ in SSRT at pre-test [*F*(3,104) = 1.85; *p* = 0.14; $\eta_{p}^{2}$ = 0.05]. As for response inhibition, while the corresponding ANOVA did not revealed a reliable effect of group [*F*(3,104) < 1; $\eta_{p}^{2}$ = 0.01], the only reliable pre–post reduction of SSRT was in the ICT group (**Table 1**). Note that there were training-related effects neither on hits nor on the RTs of Go trials^3^. Thus, training effects were only evident in the SSRT as an index of response inhibition, but not in the other variables that assess basic task performance. This is important since it shows that transfer is specific to the executive control trained process.

#### Operation Span

For the O-Span task, we considered the number of words recalled (storage capacity) and the averaged accuracy of equations (ongoing processing) multiplied, and resulting as a combined index of dual processing. For the calculation, we used a partial credit load scoring approach (PCL, Conway et al., 2005), which considered the average proportion of correctly recalled words from all set sizes, regardless of whether the set size group was perfectly recalled or not.

A one-way ANOVA on the combined scores (words recalled and equations accuracy) showed that there were no differences between the groups at pre-test [*F*(3,108) = 0.42; *p* = 0.73; $\eta_{p}^{2}$ = 0.01]. Particularly, there was a reliable pre–post enhancement in the three training groups, with the greatest effect size in the ICT group (**Table 1**). The ANOVA comparing the groups’ standardized gains failed to show reliable differences however, *F*(3,108) = 1.76; *p* = 0.15; $\eta_{p}^{2}$ = 0.04.

Finally, intrusions were also considered as a measure of updating in WM (low intrusion corresponding to successful updating). In this case, only the WMT group was able to significantly reduce the number of intrusions in the dual task (**Table 1**). The one-way ANOVA confirmed a reliable effect of group, *F*(3,108) = 2.73; *p* = 0.04; $\eta_{p}^{2}$ = 0.07. The *post hoc* comparisons showed that the only reliable difference involved the WMT and the PC groups (*p* = 0.03).

#### AX-CPT

To assess the tendency toward proactive/reactive control, we calculated the Behavioral Shift Index (BSI)^4^ introduced by Braver et al. (2009) and Chiew and Braver (2014). Larger BSIs stands for a greater tendency toward proactive control, whereas smaller BSIs indicate a tendency toward reactive control. Invalid trials, which included no responses and trials with responses times below 100 or above 1000 ms, were 6.1% out of the trial total. Eight participants were removed from the analysis because they had more than 10% of invalid trials in pre- and post-test. The four groups were comparable in BSI at pre-test [*F*(3,100) = 0.21; *p* = 0.88; $\eta_{p}^{2}$ = 0.01].

The one-way ANOVA on BSI standardized gains failed to show a group effect [*F*(3,100) = 1.60; *p* = 0.19; $\eta_{p}^{2}$ = 0.04]. Nonetheless, the pre–post analyses only showed a reliable effect in both ICT and WMT groups, which exhibited larger BSI after the training (**Table 1**, *p*s ≤ 0.01).

#### Raven’s Advanced Progressive Matrices

There were no differences between the groups at pre-test in either hit rates [*F*(3,108) = 0.44; *p* = 0.72; $\eta_{p}^{2}$ = 0.01], or reaction times [*F*(3,108) = 1.47; *p* = 0.22; $\eta_{p}^{2}$ = 0.03]. As shown in **Table 1**, however, the ICT was the only group that exhibited a pre–post increase in hit rates. The one-way ANOVA confirmed an effect of group, *F*(3,108) = 2.63; *p* = 0.05; $\eta_{p}^{2}$ = 0.06), which was mainly accounted for by the difference between the ICT and PC groups (*post hoc* comparison with *p* = 0.04). No effects were found in hit reaction times.

### Predictors for Training Improvement and Transfer

We were also interested in exploring which of the cognitive abilities tested at the baseline level predicted the magnitude of training improvement. We ran linear regression analyses with the average training slope as the outcome, and all the measures at the pre-testing stage as predictors. Only for the experimental training groups, RAPM scores significantly predicted the global training improvement (*R*^2^ = 0.12; *p* = 0.01; β = 0.86). We also looked at whether pre-test performance on the reasoning test predicted transfer gains after training. However, there was not a reliable relationship between RAPM scores before training and any of the gain scores on the transfer tasks. Reasoning scores at pre-test only predicted reasoning scores at post-test (*R*^2^ = 0.24; *p* < 0.01; β = 0.45).

Going a step further we also looked at whether the magnitude of training improvement predicted transfer gains. We ran linear regression analyses in each training group, setting the standardized gains in the different transfer tasks as the criterion and the average training slope as the predictor variable. In the ICT group, higher training improvements predicted larger gains in the relative conflict score of the Stroop task (*R*^2^ = 0.29; *p* < 0.01; β = 0.19) and larger gains in the RAPM (*R*^2^ = 0.12; *p* = 0.04; β = 0.09). No reliable regressions emerged for the WMT and the AC groups (all with *p*s > 0.15).

On the whole trained sample, the analyses showed that the level participants were able to achieve in the training activities only predicted performance in the criterion tasks; namely, conflict in Stroop (*R*^2^ = 0.12; *p* < 0.01; β = 0.13) and errors in the *n*-back task (*R*^2^ = 0.05; *p* = 0.03; β = -0.10).

### Motivation Results

In order to account for the motivational factors during training, every two sessions we asked participants about their: (i) involvement in the program; (ii) perceived difficulty of the activity levels; (iii) perceived challenge of improving over the levels; (iv) expectations for their achievement (Alonso-Tapia and de la Red Fadrique, 2007; Colom et al., 2013). We averaged all the variables across the three measurement points and explored their distribution across groups. One-way ANOVAs failed to show group differences in any of the four motivational variables: implication [(AC: *M* = 9.09; *SD* = 0.85; ICT: *M* = 9.03; *SD* = 0.94; WMT: *M* = 8.98; *SD* = 0.92); *F* < 1; *p* = 0.90, $\eta_{p}^{2}$ = 0.00]; perceived difficulty [(AC: *M* = 6.31; *SD* = 1.46; ICT: *M* = 6.11; *SD* = 1.76; WMT: *M* = 6.25; *SD* = 1.49); *F* < 1; *p* = 0.8, $\eta_{p}^{2}$ = 0.00]; perceived challenge to improve [(AC: *M* = 7.15; *SD* = 1.46; ICT: *M* = 7.31; *SD* = 1.47; WMT: *M* = 7.28; *SD* = 1.52); *F* < 1; *p* = 0.91, $\eta_{p}^{2}$ = 0.00] and expectations to improve [(AC: *M* = 7.28; *SD* = 1.58; ICT: *M* = 7.76; *SD* = 1.20; WMT: *M* = 7.94; *SD* = 1.07); *F*(2,85) = 1.91; *p* = 0.15; $\eta_{p}^{2}$ = 0.04]. Then, we calculated partial correlations controlling for group between the four motivation variables and the global training slope. We only found a modest correlation between the training slope and the perceived challenge (*r* = 0.25; *p* = 0.04), so that those participants who perceived the training as more challenging were the ones who tended to improve the most.

In order to explore whether participant’s motivation modulated training improvement, we averaged the four motivation variables and calculated a global motivation score (AC: *M* = 6.81; *SD* = 0.69; ICT: *M* = 6.99; *SD* = 0.75; WMT: *M* = 6.99; *SD* = 0.54). A one-way ANOVA showed no differences between the groups in general motivation, *F* < 1; *p* = 0.54; $\eta_{p}^{2}$ = 0.01. However, because we wanted to more precisely examine whether the motivation level was related to the participants’ training achievement, we split all the executive control trained participants by the median of the global score (*Md* = 6.95) to differentiate between high and low motivated participants. A one-way ANOVA with motivation (high and low) as the factor and global training slope of ICT and WMT participants as the dependent variable showed motivation levels to be statistically significant, *F*(1,62) = 5.55; *p* = 0.02; $\eta_{p}^{2}$ = 0.08); with high motivated participants exhibiting a higher training slope (*M* = 9.39; *SD* = 2.65) than less motivated participants (*M* = 7.82; *SD* = 2.66).

To explore whether motivation predicted transfer, multiple linear regression models were run setting the standardized gains in the transfer tasks as criterion variables, the four motivational variables measured during training as predictor variables, and considering the two training groups as a whole. The level of motivation predicted transfer to the O-Span task in the two experimental training groups (*R*^2^ = 0.15; *p* = 0.03), so that those who felt more involved (β = 0.28; *p* = 0.03) and those who perceived the training as less difficult (β = -0.31; *p* = 0.02) had larger gains after training.

Lastly, we compared the transfer gains in those participants who were highly motivated from the experimental groups (ICT: *n* = 17; WMT: *n* = 15) with those who were highly motivated in the active control group (*n* = 12). Most likely due to the small sample sizes, only a marginal statistical effect was found on the standardized gains of one of the two criterion tasks. Specifically, in the *n*-back task the WMT participants had larger gains (*M* = 0.92; *SD* = 0.99) than the ICT (*M* = 0.13; *SD* = 1.07) and the AC participants (*M* = 0.19; *SD* = 0.96), [*F*(2,31) = 2.80; *p* = 0.07; $\eta_{p}^{2}$ = 0.12].

## Discussion

The main goal of the present study was to directly compare the effectiveness of two specific process-based EFs training programs (WM and IC) in young adults. These two programs were based on the assumption of the highly influential “Unity and Diversity” model of EFs proposed by Miyake et al. (2000). The main feature of this model is that the EFs system could be partitioned into overlapping (unity) and yet distinct (diversity) components (inhibition, shifting, and WM updating). A logical conclusion drawn from the assumption of diversity is that EFs training could specifically be targeted to one of these functions with transfer effects showing some degree of specificity and commonality. The results of the present experiment generally support this assumption.

Thus, regarding the improvement in the criterion tasks – structurally similar to the trained ones – our results support the specificity of EFs training on the basis of the specific benefits observed at post-test. Only the WMT group showed pre–post enhancement in the *n*-back task (*n*-back level and errors) and only the ICT group exhibited reduced conflict scores in the Stroop task after training. Even though some previous studies have shown benefits in the Stroop task following WM training (Borella et al., 2010; Chein and Morrison, 2010; Schweizer et al., 2011), we failed to observe a reliable effect of WMT over conflict resolution. Hence, the results concerning the criterion tasks point to straightforward training-specific effects.

In relation to near transfer effects, we also observed specific training benefits for the WMT group in the non-trained WM task (O-Span). Particularly, for the O-Span task only the WMT group showed a benefit in suppressing memory intrusions; consistent with previous studies showing the relationship between high WMC and more efficient intrusions suppression in span tasks (Rosen and Engle, 1998; Turley-Ames and Whitfield, 2003; Borella et al., 2008). Similarly, the ICT group was the only group that specifically showed a benefit in response inhibition (SSRT), indicating that adaptive training in conflict resolution tasks improves performance in other tasks also thought to require conflict resolution mechanisms (for related results, see Logan and Burkell, 1986; Manuel et al., 2013; Berkman et al., 2014; Enge et al., 2014; Dovis et al., 2015). Together with the results found with the criterion tasks, the near transfer results also support the idea that training on either WM or IC leads to specific performance benefits in tasks related to the training (Simons et al., 2016).

However, and despite this specificity, the two EFs-trained groups also showed some common features regarding near transfer effects. Thus, both WMT and ICT groups improved dual performance (Equations accuracy × Words recalled) in the O-Span task [related findings of improved complex span scores have been reported after simple, complex span and visual search training, (Harrison et al., 2013); rehearsal strategy training (Turley-Ames and Whitfield, 2003) or task-switching training (Karbach and Kray, 2009)], suggesting that dual tasking may require both WM capacity and IC mechanisms (Towse et al., 2000; Smith et al., 2001; Unsworth, 2010; Chein et al., 2011). Hence, IC seem to be demanded not only in the training activities practiced by our ICT group but also in the WM updating tasks that required suppression of irrelevant information and that were extensively practiced by the WMT group. This might be indicating the relationship between WM and IC at the behavioral level, and be suggestive of the degree to which trained and transfer processes may overlap in their underlying neuro-cognitive networks. Kane and Engle (2002) proposed that dorsolateral prefrontal cortex could play a role in WM capacity in contexts providing potential interference (and requiring attentional control). Conway et al. (2003) and Gray et al. (2003) agree that in WM span tasks regions in the prefrontal cortex are activated when an executive control mechanism is recruited to reduce interference during the maintenance and manipulation of information.

It is, however, puzzling that we also observed a training effect for the active control group in the O-Span task, which did not differ from that obtained by the WMT group. Note that, although the AC group did not increase the cognitive load over the training levels, we used activities that involved increasing difficulty by augmenting the speed of processing. Thus, as also predicted, it is possible that the positive effect for this control group stemmed from the overarching time-limited nature of the tasks. Increasing speed of processing could have led to more efficient processing and maintenance in WM that would result in better performance in the O-Span task. Unsworth et al. (2009) reported a negative correlation between processing speed and WM maintenance, suggesting that participants who processed quickly recalled more items that those who worked slowly. Similarly, faster speed processing has been proposed to reduce the possibility of items being forgotten, and less time for rehearsing or refreshing processes (Towse et al., 2000; Hudjetz and Oberauer, 2007; Unsworth et al., 2009).

Regarding far transfer effects, we also found common and diverse features in our trained groups. We included two tasks (AX-CPT and RAPM) that did not directly capture WM or IC: the AX-CPT was used to explore whether training effects might change the control strategy used by the participants, and Raven’s matrices to explore whether WM and IC training transferred to a more general complex domain such as abstract reasoning. The AX-CPT is widely used to explore the dynamic adjustment of cognitive control strategies and it has shown to be very sensitive to individual differences in cognitive control (Braver et al., 2009; Burgess et al., 2011; Braver, 2012). Proactive control requires goal maintenance and is related to paying attention to contextual cues in order to effectively solve interference while keeping the monitored cues in mind (Rush et al., 2006). In this version of the task, the use of a proactive control strategy was encouraged since the context was highly predictive (the A cue precedes the X target in 70% of the trials); hence, a control mode that involves sustained maintenance of task-relevant information would lead to a high success rate, albeit it would lead to errors in trials where the cue was A but the probe was not X (AY trials; 10% of the trials). Thus, enhanced proactive control is expected to increase AY errors and reduce BX errors, with the BSI tending to larger values since the cue in BX trials does not signal a “yes” response. Usually, because it is the most efficient strategy, young adults exhibit behavioral performance and brain activity (sustained lateral PFC activation) consistent with a predominant proactive control strategy (Braver, 2012; Morales et al., 2013).

Interestingly, results from our experiment regarding the BSI (an index signaling changes toward proactive control) in the AX-CPT suggested a higher reliance on proactive control for WM and IC trained participants compared to active and passive control groups. Previous studies have already reported the malleability of cognitive control mechanisms engaged in the AX-CPT due to experience-based conditions such as bilingualism (Morales et al., 2013, 2015) or different kinds of training interventions: task-strategy training made older adults (Paxton et al., 2006) and people with schizophrenia (Braver et al., 2009; Edwards et al., 2009) more prone to engage in proactive control; indeed, more similar to adults-like performance than before training. Previous studies have also reported proactive shifts in cortical regions as the lateral PFC after strategy (Braver et al., 2009) and IC training (Berkman et al., 2014), suggesting the possibility that the lateral PFC might serve to anticipate upcoming control demands across a range of executive control domains. Our results replicate and extend these findings by showing behavioral shifts toward proactive processing in both ICT and WMT (even though numerically larger in the WMT group), suggesting again some common executive resources for inhibitory and WM processes.

In contrast, the results of the non-verbal reasoning (RAPM) task showed some degree of specificity. Specifically, we observed a benefit for the ICT group but not for the WMT group. The question of whether cognitive training could improve fluid intelligence is a recurrent controversial area of research with considerable number of studies reporting data against (Shipstead et al., 2012; Melby-Lervåg and Hulme, 2013) and in favor of it (Morrison and Chein, 2011; Au et al., 2014). Results of our ICT group join others showing better reasoning performance after training. Karbach and Kray (2009) reported improved performance in a composite measure of reasoning after four sessions of task-switching training in children, young and older adults compared to an active control group (Karbach and Kray, 2009). Similarly, Rueda et al. (2005) found benefits in a measure of reasoning after 5 and 10 days (Rueda et al., 2012) of executive-control training in pre-school children when compared to control groups (but see Thorell et al., 2009 and Enge et al., 2014 for failures to show such positive effects).

However, our WMT group did not show benefits in abstract reasoning. Although fluid intelligence and WM share common variance (Colom et al., 2004; Oberauer et al., 2005; Friedman et al., 2006; Harrison et al., 2015) and EFs have been related to reasoning operations (Dempster and Corkill, 1999; Engle and Kane, 2004; Jarosz and Wiley, 2012; Shipstead et al., 2015) it is possible that our participants did not reach the level of difficulty needed to show far transfer. In support of this interpretation, the results of the regression analyses showed that training improvement only predicted transfer to abstract reasoning for the ICT group, which suggests that the training levels achieved by the WMT group did not reach high enough demand levels to promote transfer. Previous studies reporting positive training effects have normally used single but highly demanding tasks, such as the dual *n*-back task (Jaeggi et al., 2008, 2010, 2013) and/or participants attained high levels of performance over training, such as *n*-back levels of over 3 (Jaeggi et al., 2008, 2013). Note that, in average, our WMT participants reached an *n*-back level below three and performed a single *n*-back task. Hence, and considering the fact that we trained more than one task, it is possible that the level of difficulty was below that needed to show far transfer effects with WM training.

Together, the observed transfer effects allow us to claim that it was the ICT group that showed the most consistent pattern of enhanced performance across tasks. While there might be more than a single reason behind this finding, we favor the idea that the benefit for the ICT group is not related to differences in cognitive demands or motivational aspects between the two training programs. As previously noted, the training levels achieved by the WMT group could have not been demanding enough to lead to stronger overall transfer.

An additional and interesting point addressed in the present work was to look at individual differences regarding training and transfer effects. This is an issue that remains to be explored in deep (Könen and Karbach, 2015). In line with previously reported studies (Bürki et al., 2014), we have found that abstract reasoning was a meaningful predictor of training improvement, indicating that people with higher reasoning scores benefited more from training. Furthermore, training improvement constituted a relevant predictor of transfer to the criterion tasks for the two experimental trained groups, and particularly in the case of the ICT group a predictor for transfer to reasoning and conflict reduction. This pattern of results highlights the importance of considering individual differences before training because they might influence how well they do during training and how much benefit they can take from it (Könen and Karbach, 2015).

In addition to the more important theoretical issues related to brain plasticity and transfer, a secondary aim of our study was methodological in nature. Previous training studies have been criticized for the suitability of the control conditions, for not considering motivational factors, or for the use of single training tasks (Jaeggi et al., 2013; Redick et al., 2013; Melby-Lervåg et al., 2016). In our study, we took these factors into account by using different tasks to train the target processes (and to increase the probability of generalization), by introducing two different control conditions (active and passive), and by considering motivational variables associated with training. Thus, the active control group engaged in tasks essentially requiring processing speed (for related approaches see Goldin et al., 2014; Lawlor-Savage and Goghari, 2016), in order to keep participants’ motivation and engagement similar to those from the experimental groups. Importantly, all trained participants (including the AC group) showed a meaningful improvement in the specifically trained process (IC, WM and processing speed). In fact, the AC group showed larger training slopes than the two experimental groups (**Figure 1**). Note again that control activities were mainly perceptual and successive levels did not engage greater executive load but only faster responses with a low constant cognitive effort.

Also of relevance, the motivation questionnaire revealed similar levels of implication, perceived difficulty, perceived challenge and expectations during training for the three trained groups, indicating that transfer differences among the groups were not due to differences in motivation or perceived difficulty. Interestingly, while motivation cannot easily account for the differences between the training and control groups, it was a factor that predicted training improvement in the experimental groups, so that highly motivated participants – those that were more involved and perceived the training as less difficult – showed larger improvements across the training sessions than less-motivated participants. Thus, and consistent with previous studies, motivation this result highlights the importance of considering individual motivation through training, since it is related to greater improvements that could result in greater transfer effects (Mather and Carstensen, 2005; Jaeggi et al., 2013; Katz et al., 2014). Apart from motivation, it would be of interest in future studies the inclusion of additional self-reporting assessments regarding individual differences in beliefs about the fixed or malleable nature of cognition (Jaeggi et al., 2013), expectancy and perceived improvement (Boot et al., 2011).

It must be noted that the present research is not without limitations. First, and despite the specific benefits found in the within-group comparisons, the lack of group effects in some of the standardized gain measures suggests caution in the interpretation of the results. Null effects in the gain comparisons may reflect the lack of statistical power but also inflated variability among groups. In a recent meta-analysis, Melby-Lervåg et al. (2016) established that training studies with large effect sizes normally included small sample sizes (less than 20 participants per training condition) and untreated (passive) control groups, which produces biases toward significant – but low powered – results (Enge et al., 2014; Melby-Lervåg et al., 2016). In the present study, we used samples that were all over 20 participants per condition and we included a passive control group as well as an active control group. Second, the present training schedule covered 2 weeks, which is in the lower end of the range of the current training studies (from 2 to 14 weeks; Morrison and Chein, 2011). Hence, it is yet to be explored the magnitude of the transfer effects when training is extended over a longer period of time. Similarly, our study is blind regarding possible long-term effects since we did not follow them up in time. Future studies should address this issue because the value of training interventions essentially relies on the durability of training-induced results. Finally, we recognize that transfer effects in studies with young healthy samples are limited as long as they might be optimally functioning at pre-testing, leaving not enough room for meaningful improvements with training. Hence, studies with children and older adults could be more sensitive to training-related changes than studies with young people (Kelly et al., 2014; Spencer-Smith and Klingberg, 2015; Weicker et al., 2016).

In closing, and despite the existing limitations, our results lead us to suggest that executive-control training may modulate cognitive abilities in young people. The malleability of EFs challenges the long-standing assumption that cognitive abilities remain fixed over time. Training cognition is not a new concept (Jolles and Crone, 2012; Boot and Kramer, 2014; Schubert et al., 2014), but the idea that training and experience can generalize to tasks and domains beyond those trained is still controversial. In this sense, our results, while being modest and at the task level – rather than at the construct level – are promising and support substantial plasticity of cognitive control mechanisms by means of training. Interestingly, the results also suggest that there is some specificity in the consequences of the trained processes so that transfer occurs only when the specific trained process is tapped by the transfer task and domain. This opens the possibility that training in applied settings may be specific to the process needed for a specific domain, or to the impaired process due to deficient brain functioning. Also, this is suggestive of setting the ambitious goal of exploring the potential benefit of executive control training for everyday activities (Simons et al., 2016). Before this, further research would need to address the potential effects of executive-control training over brain structure and dynamics. Analyzing structural and functional brain profiles may provide further insight into why specific interventions may be more successful for certain individuals, and help characterize the overlap between training tasks and tests that show training-related transfer.

## Author Contributions

This work is part of the thesis dissertation of the first author (MM). The authors developed the concept of the study together. MM contributed to the design of training tasks, data collection and analyses, and manuscript writing. MB in collaboration with M. R. Rueda designed the online program PEC-UGR. MB and CG-A supervised the process of accomplishing the study, and wrote, reviewed and approved the final version of the manuscript.

## Conflict of Interest Statement

The authors declare that the research was conducted in the absence of any commercial or financial relationships that could be construed as a potential conflict of interest.

## Footnotes

1 The Cognitive Training Program (PEC-UGR) has been developed by the collaboration between professors M. Teresa Bajo and M. Rosario Rueda from the School of Psychology of the University of Granada.

2 Proactive control refers to an “early selection” control mode that anticipates and prevents interference before it occurs, while reactive control implies a “late correction” strategy that detects and solves interferences once it is already present (Braver et al., 2009; Braver, 2012; Morales et al., 2013). The AX-CPT is a sensitive and reliable experimental task widely used to explore individual differences in the use of proactive and reactive control strategies (Braver, 2012; Chiew and Braver, 2014). Hence, we used it to assess whether the participants’ control mode changes with training. While young adults tend to rely on a proactive control strategy while performing the AX-CPT (Braver et al., 2009; Morales et al., 2013), training could somehow modulate the way they faced the task, which systematically required goal maintenance, interference detection and conflict resolution.

3 No reliable differences in pre–post effects were observed on hits (*p*s > 0.33) or RTs (*p*s > 0.56) in Go trials for the ICT, WMT or AC groups. The PC group showed a worse performance with a lower hit rate [*M*_pre_ = 95.71, *SD*_pre_ = 4.76; *M*_post_ = 91.77, *SD*_post_ = 4.56; *F*(1,21) = 8.06; *p* = 0.01; $\eta_{p}^{2}$ = 0.27]; and slower RTs [*M*_pre_ = 753.21, *SD*_pre_ = 156.80; *M*_post_ = 832.54, *SD*_post_ = 168.07; *F*(1,21) = 11.75; *p* = 0.00; $\eta_{p}^{2}$ = 0.35] in the post-test.

4 This index is based on the formula (AY - BX)/(AY + BX) for errors and reaction times. Trials where errors were equal to 0 were corrected [(errors + 0.5)/frequency of trials + 1].
